# Bioavailable iron titrations reveal oceanic *Synechococcus* ecotypes optimized for different iron availabilities

**DOI:** 10.1038/s43705-022-00132-5

**Published:** 2022-07-01

**Authors:** Naomi E. Gilbert, Gary R. LeCleir, Robert F. Strzepek, Michael J. Ellwood, Benjamin S. Twining, S. Roux, C. Pennacchio, Philip W. Boyd, Steven W. Wilhelm

**Affiliations:** 1 Department of Microbiology, The University of Tennessee, Knoxville, TN 37996 USA; 2 Institute for Marine and Antarctic Studies, University of Tasmania, Hobart, TAS 7004 Australia; 3 Research School of Earth Sciences, Australian National University, Canberra, ACT Australia; 4 Bigelow Laboratory for Ocean Sciences, East Boothbay, ME USA; 5 DOE Joint Genome Institute, Lawrence Berkeley National Laboratory, Berkeley, CA USA; 6 Present Address: Australian Antarctic Program Partnership (AAPP), Institute for Marine and Antarctic Studies, University of Tasmania, Hobart, TAS 7004 Australia

**Keywords:** Water microbiology, Transcriptomics

## Abstract

The trace metal iron (Fe) controls the diversity and activity of phytoplankton across the surface oceans, a paradigm established through decades of in situ and mesocosm experimental studies. Despite widespread Fe-limitation within high-nutrient, low chlorophyll (HNLC) waters, significant contributions of the cyanobacterium *Synechococcus* to the phytoplankton stock can be found. Correlations among differing strains of *Synechococcus* across different Fe-regimes have suggested the existence of Fe-adapted ecotypes. However, experimental evidence of high*- versus* low-Fe adapted strains of *Synechococcus* is lacking, and so we investigated the transcriptional responses of microbial communities inhabiting the HNLC, sub-Antarctic region of the Southern Ocean during the Spring of 2018. Analysis of metatranscriptomes generated from on-deck incubation experiments reflecting a gradient of Fe-availabilities reveal transcriptomic signatures indicative of co-occurring *Synechococcus* ecotypes adapted to differing Fe-regimes. Functional analyses comparing low-Fe and high-Fe conditions point to various Fe-acquisition mechanisms that may allow persistence of low-Fe adapted *Synechococcus* under Fe-limitation. Comparison of in situ surface conditions to the Fe-titrations indicate ecological relevance of these mechanisms as well as persistence of both putative ecotypes within this region. This Fe-titration approach, combined with transcriptomics, highlights the short-term responses of the in situ phytoplankton community to Fe-availability that are often overlooked by examining genomic content or bulk physiological responses alone. These findings expand our knowledge about how phytoplankton in HNLC Southern Ocean waters adapt and respond to changing Fe supply.

## Introduction

Nutrient availability exerts a fundamental influence on microbial communities inhabiting surface oceans, constraining both oceanic and atmospheric biogeochemical cycling [1]. Specifically, the trace metal iron (Fe) influences carbon fixation (*via* primary productivity) and carbon export to the deep ocean [2, 3]. Fe-limitation of phytoplankton has been studied for decades, resulting in the discovery of high nutrient, low chlorophyll (HNLC) regions that account for ~40% of the world’s surface oceans [4]. Most field studies capture the community-response of Fe addition on the physiology of phytoplankton such as diatoms: these observations form our foundation of knowledge for how Fe impacts photosynthetic organisms [5, 6]. Indeed, the discovery of low-Fe adapted diatoms provided insight into the ability of specific phytoplankton to cope with chronic Fe-stress. Specifically, it highlighted how they acquire Fe, decrease cellular Fe requirements [7], and substitute enzymes that require large amounts of Fe with more parsimonious ones [8–10]. However, there is evidence that prokaryotic phytoplankton, the cyanobacteria [11], also exhibit adaptations to Fe-limitation. Despite widespread Fe-limitation in the surface ocean, *Prochlorococcus* and *Synechococcus* are abundant and contribute significantly to global primary production [12, 13]. These picophytoplankton have adapted to nutrient-poor conditions through genomic streamlining [14] and by maintaining small cell sizes, which (for spherical cells) increases their surface-to-volume ratios [15]. Some cyanobacteria also alter their cellular and photosynthetic structures, lose genes encoding high Fe proteins [16] and in some cases may use sophisticated Fe acquisition mechanisms [17, 18] to adapt to Fe-limited conditions.

There is a rich history of research into factors that select for different phytoplankton species in aquatic systems [19]. Genomic analyses have pointed to *Synechococcus* (and *Prochlorococcus*) ecotypes that are correlated with regions of differing Fe availabilities [20–23]. However, when comparing genomes to distinguish low-Fe (HNLC) and high-Fe (coastal) ecotypes of *Synechococcus*, observations of genome-encoded functions specific to adaptations to differing Fe-regimes do not fully correspond to the respective niches they occupy [21]. The hypothesis that local conditions shape microbial communities at a more complex scale than simply high-Fe *versus* low-Fe needs to be investigated to generate accurate predictions of how oceanic microbial communities respond to nutrient limitation. Furthermore, given the contributions of cyanobacteria to global carbon cycles and the paucity of field-based studies investigating gene-level responses to Fe availability, it is important to disentangle how this group of phytoplankton respond to Fe availability. Most importantly, it is critical to test the response(s) of putative Fe-ecotypes beyond correlative observations.

The Southern Ocean Time Series (SOTS) is located in the Sub-Antarctic Zone (SAZ) [24, 25], a region of the global ocean that plays a critical role in CO_2_ uptake and its subsequent export to the deep ocean [26]. While primary productivity in this region is typically limited by Fe availability seasonally [2, 27], the region encounters periodic inputs of trace metals and these processes are expected to change with new climate trajectories [28]. Thus, SOTS represents an ideal location for the assessment of phytoplankton community response to changes in Fe availability.

We examined the response of phytoplankton to manipulated Fe availability—both increasing and decreasing - within short-term, on-deck bottle-incubations during a Spring (March 2018) expedition at SOTS. High-throughput sequencing of the total microbial mRNA pool (metatranscriptomics) was done for the entire microbial community but presented here with a focus on marine *Synechococcus* spp. We initially hypothesized that Fe additions would stimulate total community productivity, and that Fe removal would lead to an Fe-stress response by all microbial members. During an examination of *Synechococcus* spp. transcripts, we observed a more complex response, suggesting the presence of both high and low-Fe adapted strains residing within the SOTS surface waters. This study also establishes an experimental framework to employ metatranscriptomics to characterize the entire phytoplankton community across a gradient of Fe availabilities in a manner that allows for a clearer interpretation of *in situ* population-level physiology and dynamics.

## Methods

### Cruise details, sample collection and iron incubation

Iron amendments and in situ water column sampling were conducted at SOTS onboard the *R/V Investigator* during March 2018 using trace-metal-clean conditions [29]. In situ samples were collected prior to sunrise from 15 m depth using 12 L Niskin bottles on a CTD rosette at 47° 00’ 01.6“S, 142° 01’ 14.7“E (March 5th), 46° 59’ 52.6“S, 141°59'57.6“E (March 7th), and 47° 00’ 01.8“S, 142° 00’ 07.9“E (March 9th). Concentrations of nitrate (NO_3_), phosphate (PO_4_), silicate (Si), nitrite (NO_2_), and ammonium (NH_4_) were determined for unfiltered samples using a Seal AA3 segmented flow system following previous procedures [30]. Two Fe incubation experiments were performed: one on March 5th, 2018 (GRW1) and one on March 14th, 2018 (GRW2). Homogenized seawater collected from a trace-metal clean pump at 5 m was allocated into 2 L Nalgene™ bottles. For GRW1, either 0.25 nM, 0.5 nM, 1.0 nM, or 2.5 nM of either desferrioxamine-B (DFB) or Fe chloride (FeCl_3_) was added to chelate Fe or increase Fe, respectively. For GRW2, the procedure was repeated except bottles were amended with either 1.25 nM, 5.0 nM, or 12.5 nM DFB, or 2.5 nM FeCl_3_ as Fe-limitation conditions (based on in situ chlorophyll a fluorescence and Fv/Fm) appeared to wane [31]. An control was included for each experiment and T = 0 h sample was also collected (in situ, 5 m). Bottles were incubated at ~33% incident irradiance and ambient surface temperature (~11^o^ C). After 72 h, bottles were sampled for chlorophyll *a*, photosynthetic health (F_v_/F_m_), and total RNA. See the Supplemental Material for further details.

### RNA extraction, preprocessing, and sequencing

RNA was extracted with a phenol-chloroform based approach using a publicly available protocol [32]. Residual DNA was removed using the Turbo DNA-*free*™ kit (Ambion®). Libraries were prepared and sequenced by the DOE Joint Genome Institute (JGI) using the low-input protocol for total RNA. Briefly, ribosomal RNA was depleted using the Illumina® Ribo-Zero rRNA Removal Kit (Bacteria) and sequenced using paired-151 nt reads on the Illumina Novaseq S4 platform. Biological duplicates for all treatments were sequenced for incubations except for one control bottle from both GRW1 and GRW2 due to insufficient RNA.

### Bioinformatic analyses

Raw read filtering and trimming was done using BBDuk v38.67 and BBMap v38.84 from the BBtools suite of packages [33]. Trimmed reads from all 18 incubation samples for GRW1, including the T = 0 h in situ sample, were co-assembled using MEGAHIT v1.2.9 [34]. Open reading frames (ORFs) were called using MetaGeneMark v3.38 [35] and reads mapped to the assembly with BBMap v38.84 were tabulated using featureCounts v2.0.0 [36]. For direct comparison of the in situ samples, these trimmed reads were also mapped to the GRW1 assembly. Read counts were normalized using the transcripts-per-million (TPM) approach [37]. Data visualization was performed using ggplot2 in the R statistical platform [38, 39]. The Pheatmap R-package was also used for hierarchical clustering and heatmap generation of functional genes [40]. Raw and processed data used in this study are publicly available through the JGI Data Portal (https://data.jgi.doe.gov) under the Project ID numbers 1260738 and 1260735.

Taxonomic composition was probed using the DNA-directed RNA polymerase beta subunit (*Rpb1/RpoB*) marker gene [41]. Briefly, GRW1 proteins were queried against a reference Rpb1/RpoB database using DIAMOND BLASTp [42]. Hits were clustered at 95% aa similarity using CD-HIT [43] and queried against the NCBI non-redundant database using DIAMOND BLASTp to retain only Rpb1/RpoB. Hits ≥1200 aa were aligned to reference Rpb1/RpoB proteins (Supplementary Table 1) in MEGA7 [44] using ClustalW [45]. For further exploration of cyanobacterial RpoB phylogenies, unclustered protein sequences were used to construct a separate tree containing all eukaryotic and prokaryotic references, with additional *Synechococcus* RpoB reference sequences (Supplementary Table 2). The separate maximum-likelihood trees were constructed in PhyML [46], and hits <1200 aa were placed on the tree using pplacer [47] and visualized/annotated using iTOL v.4 [48].

ORFs were functionally annotated using eggNOG-mapper v2.0.1 [49] and FeGenie [50]. Taxonomic annotation of ORFs was done using the “best LAST hit” procedure by the IMG pipeline [51, 52]. Only genes annotated as *Synechococcus* using this tool and confirmed to be of cyanobacterial origin *via* eggNOG-mapper*’s Best Tax Level*, were referred to as “*Synechococcus-*like transcripts”. To investigate the numerous hits (763) annotated as “*oprB*-like” porins (Supplementary Table 3), translated sequences were aligned separately to Slr1908 protein sequences identified as “Fe-uptake porins” in cyanobacteria [53] or the NCBI non-redundant database (downloaded January 2022) using DIAMOND BLASTp [42], with an e-value threshold of 1e-10 retaining 5 hits per query (Supplementary Table 4). The ferritin phylogenetic tree was constructed using the same approach outlined for RpoB/RPB1 using *Synechococcus* isolate ferritin sequences downloaded from NCBI (June 2021). Putative *Synechococcus* ferritin sequences >190 aa were used within the base tree (Supplementary Table 5).

Competitive read recruitment to two representative *Synechococcus* genomes was done to expand upon the observations from metatranscriptome assemblies, and to better resolve differences in transcript abundance patterns that may be driven by differences in gene copy numbers. Coding sequences from sequenced genomes of *Synechococcus* sp. CC9311 (CP000435.1) and *Synechococcus* sp. BL107 (NZ_DS022298.1) were downloaded from NCBI on June 12th, 2021, chosen based on high similarity of *rpoB* and ferritin-like sequences within the assembly (Supplementary Figs. 2 and 5) representing isolates from divergent trophic environments (coastal vs open-ocean). Trimmed reads from the Fe-incubations and in situ metatranscriptomes were mapped to both genomes simultaneously with 85% similarity fraction over 90% read length in CLC Genomics Workbench v.21.0.4 (Qiagen), retaining non-redundantly mapped reads. See the Supplemental Material for further details on bioinformatic analysis.

### Statistical analyses

Differential transcript abundance analysis (*i.e.,* “Differential Expression”) and principal components analyses (PCA) were computed on the ORFs from the combined assembly using DeSeq2 v1.28.1 within R [39, 54]. For differential transcript analysis, fold change and adjusted *p* values (p_adj_) were calculated for the following comparisons within the GRW1 incubation: 2.5 nM Fe/2.5 nM DFB, 2.5 nM Fe*/*Control, and 2.5 nM DFB/ Control. To compare transcript abundances of genes of interest between DFB-added incubations, Fe-added incubations, and surface in situ samples, ordinary one-way ANOVAs or Kruskal-Wallis tests were performed. Post-hoc multiple comparisons were adjusted with Tukey’s HSD (ANOVA post-hoc) or Dunn’s test (Kruskal-Wallis post-hoc). For comparisons between normalized transcripts of genes of interest grouped under DFB-added or Fe-added treatments, either unpaired two-tailed *t* tests, unpaired *t* tests with Welch’s correction, or Mann–Whitney tests were performed when appropriate. See Supplementary Methods for details on statistical analyses.

## Results

### Physicochemical status

The Southern Ocean Time Series (SOTS) is located along the northern edge of the sub-Antarctic zone close to the subtropical front (STF). Surface water temperature ranged between 11 and 13 °C, salinity between 34.5 and 34.9, NO_3_ between 8.35 and 12.7 µmol L^−1^ and Si between 0.6 and 0.8 µmol L^−1^, over an 11 day sampling period, consistent with northern sub-Antarctic zone water properties [53]. Dissolved Fe concentrations in the upper 50 m ranged between 0.15 and 0.35 nmol L^−1^. The variability in dissolved Fe concentrations mirrored salinity and temperature in the upper water column. Below the surface, Sub-Antarctic Mode Water (SAMW) and Antarctic Intermediate Water (AAIW) were present between about 200–400 m, and 600–1200 m, respectively.

### Community physiological and transcriptional response to Fe availability

**Fig. 1:**
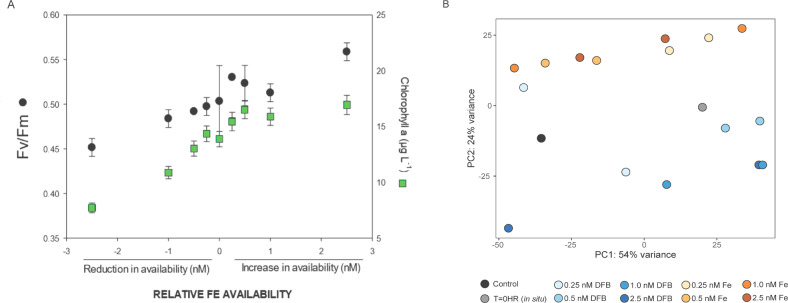
Bulk community response to on-deck incubations either reducing available Fe (DFB-added) or increasing available Fe (FeCl_3_-added) after 72 h. **A** Total phytoplankton biomass estimates (chlorophyll *a* concentrations, green boxes) and photosynthetic efficiency (F_v_/F_m_, ratio of variable fluorescence to maximal fluorescence, black circles) as a function of relative Fe availability. Results are shown as the means ± error of technical duplicates, averaged between duplicate bottles. **B** Principal component analysis (PCA) displaying PC1 against PC2 of total community gene transcript abundance patterns, color coded by treatment (blue = DFB-added, orange = Fe added). The black circle is the control bottle and gray circle is the T = 0 h in situ sample. Each bottle duplicate is plotted, with the exception of the control.

The changes in relative Fe availability generated in our experiments were positively correlated with measures of photosynthetic biomass (chlorophyll *a*) and photosystem II photochemical efficiency (F_v_/F_m_, Fig. 1A). Relative to the control, phytoplankton biomass increased with added FeCl_3_ and decreased with added DFB (Fig. 1B). For the second incubation (GRW2), Fe-addition stimulated increases in F_v_/F_m_ while DFB addition reduced bulk chlorophyll *a* and F_v_/F_m_, although these values were similar between the 12.5 nM DFB and 5.0 nM DFB-added treatments (Supplementary Fig. 1). For GRW1 specifically, the effects of these treatments on gene transcript abundance between +Fe and +DFB resulted in distinct patterns, except for one 0.25 nM DFB treatment (Fig. 1B). Despite inherent bottle variability present, the genetic signatures driving the separation of +DFB and +Fe were apparent and thus further investigated.

### Taxonomically-resolved community response to Fe

**Fig. 2:**
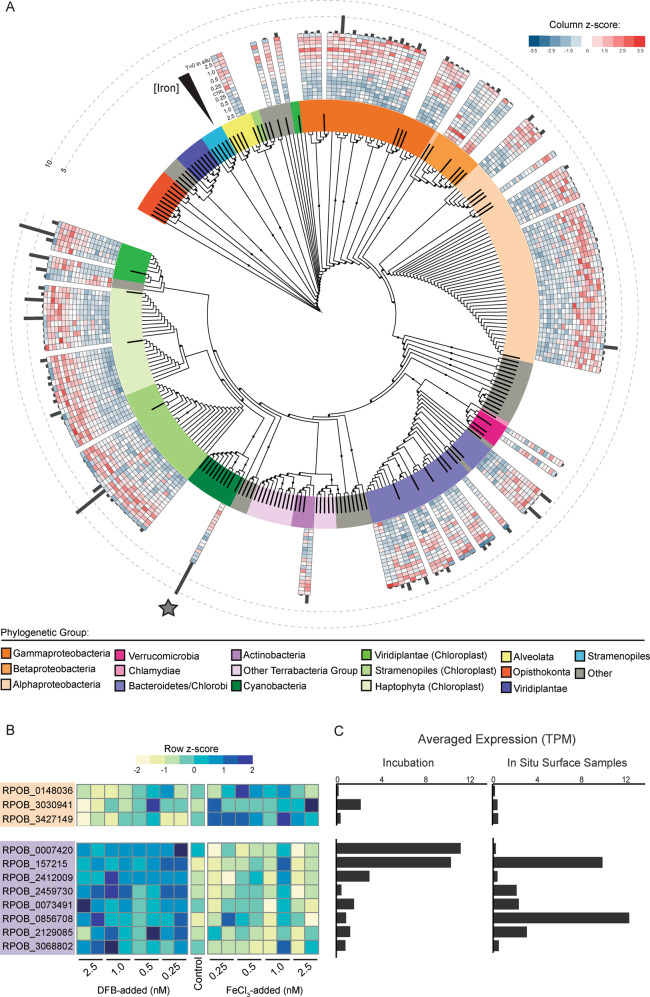
Taxonomically resolved community activity in response to Fe availability using *rpoB*/*rpb1* as a marker gene. **A** Phylogenetic placement of translated candidate *rpoB/rpb1* clustered at 95% amino acid identity across eukaryotes (blue branches) and prokaryotes (red branches), color coded by broad phylogenetic group. Reference proteins used to construct the tree are represented as black bars, and black dots are bootstrap values >0.5. Normalized transcript abundance (transcripts-per-million, TPM) levels are shown in the heatmap on the outer rings and are scaled (z-score, [Observed TPM – mean TPM]/standard deviation) for each tree candidate, going from low Fe to high Fe from the inner to outer ring. Black bars on the outermost ring represent in situ [T = 0 h, March 5th (5 m), March 5th (15 m), March 7th (15 m), March 9th (15 m)] averaged TPMs. The gray star represents the placement of a candidate in the phylum Cyanobacteria. **B** Un-clustered cyanobacterial-like *rpoB* transcript abundance (TPM, z-score) against Fe availability within the incubations. Orange block = sequences phylogenetically clustered with the *Synechococcus* sp. CC9311 reference RpoB. Purple block = sequences phylogenetically clustered with the *Synechococcus* spp. HB1133, BL107, and CC9902. The full phylogeny is shown in Supplementary Fig. 1**C** Averaged transcript abundance values (TPM) within the Fe incubation and the in situ samples, corresponding to each candidate *rpoB* row.

The DNA-directed RNA polymerase beta subunit genes (*rpoB/rpb1*), used previously as a housekeeping transcript for eukaryotes and prokaryotes within metatranscriptomes [41, 55, 56], was targeted to resolve taxonomic structure. Phylogenetic placement of candidate *rpoB/rpb1* translated sequences (clustered at 95% amino acid similarity) responsive to Fe treatments show assignment to groups ranging from heterotrophic bacteria and photosynthetic eukaryotes (Fig. 2A). Cyanobacterial RpoB was most closely related to *Synechococcus* and *Prochlorococcus* references (Fig. 2A and Supplementary Fig. 2). These candidates showed high relative transcript abundance across the in situ surface samples and higher representation within +DFB treatments relative to +Fe (Fig. 2A). However, closer examination of un-clustered, putative *Synechococcus* RpoB (Supplementary Fig. 2) sequences show the presence of individual RpoB in both high-Fe and low-Fe treatments (Fig. 2B). When phylogenetically resolved, the high-Fe RpoB were most similar to *Synechococcus sp*. CC9311 reference, and low-Fe responsive RpoB similar to *Synechococcus spp*. HB1133, BL107, and CC9902 references (Supplementary Fig. 2). Analyses of chlorophyll biosynthetic genes for algal eukaryotes and prokaryotes (Supplementary Fig. 3, Supplementary Table 6) demonstrated *Synechococcus* sp. CC9902-, BL107-, and WH8016-like transcripts were higher in low-Fe treatments and in situ (Supplementary Fig. 3). Conversely, transcripts annotated as chlorophyll biosynthetic genes from Chlorophyta, Streptophyta, *Synechococcus* CC9311 and *Prochlorococcus* were higher under high-Fe conditions (Supplementary Fig. 3).

### Functional response of *Synechococcus* to Fe availability

#### Differential transcript abundances of Fe cycling genes

**Fig. 3:**
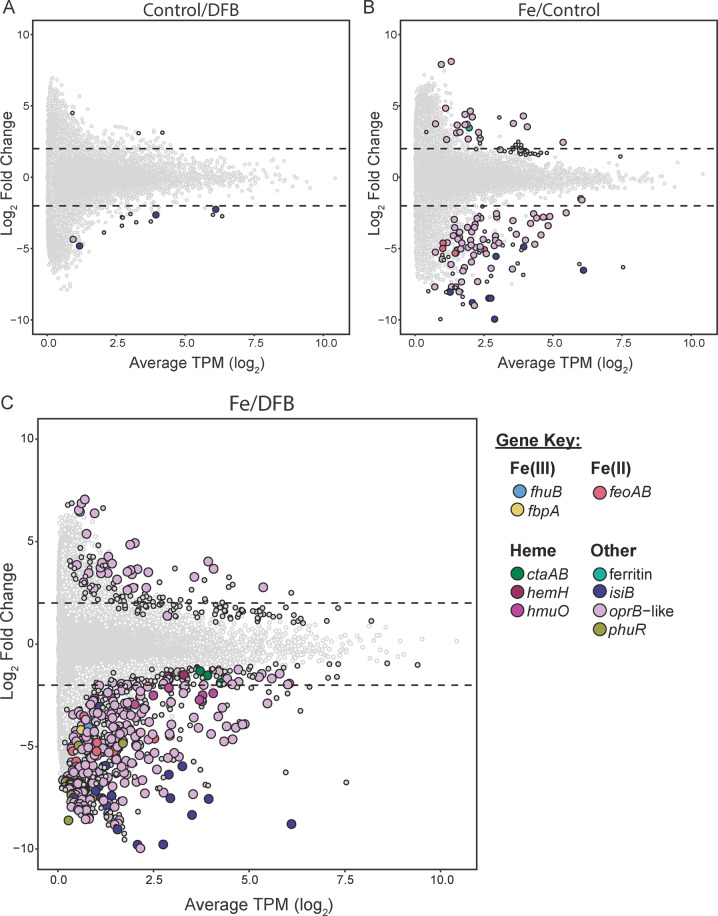
*Synechococcus* Fe-responsive genes with significantly different transcript abundances under high Fe *versus* low Fe conditions. Genes with significantly different transcript abundances (p_adj_ < 0.1) are outlined in black. The smaller, gray circles are genes in the “other” category (not Fe-related). The dashed lines show -2 and +2 log_2_ fold change thresholds. **A** Control *versus* 2.5 nM DFB **B** 2.5 nM FeCl_3_ versus Control **C** 2.5 nM FeCl_3_ *versus* 2.5 nM DFB.

To investigate functional responses to Fe availability, differential transcript abundance analyses were performed with *Synechococcus-*like genes within the assembly between high-Fe (2.5 nM Fe), low-Fe (2.5 nM DFB), and control incubations (Fig. 3A–C, Supplementary Table 7). Results for all genes (including non-*Synechococcus*-like) are in Supplementary Table 8 as well. Comparing high-Fe/low-Fe treatments generated the most genes with significantly different transcript abundances (p_adj_ ≤ 0.1), whereas the control/low-Fe analysis had the fewest (Fig. 3A–C). Because most genes were associated with Fe metabolism, we focused on this functional group, although genes within nitrogen/carbon metabolism and photosynthesis also had significantly different transcript abundances (Supplementary Table 7).

Transcripts for Fe(III), Fe(II), and putative heme-cycling genes were significantly decreased when Fe was added (Fig. 3B and C). It is possible that *phuR*, a *“Heme/hemoglobin uptake outer membrane receptor*” [57], targets non-heme sources, and so it has been denoted as an “organic iron complex“ transporter in the “Other” category (Fig. 3). Notably, 268 genes annotated as *oprB*-like Fe/carbon uptake porins were identified in the high-Fe/low-Fe analysis (Fig. 3C). Further analyses of all *Synechococcus* assigned *oprB-*like genes indicate 763 unique sequences with high sequence similarity to experimentally confirmed Fe-selective porins (Supplementary Table 9) in *Synechocystis* [53]. Comparison to all proteins within the NCBI non-redundant database also indicate numerous hits to “iron uptake porin” and/or proteins with the cl41527 conserved domain (“por_somb Superfamily/iron uptake porin”, Supplementary Table 4). Other top hits included “OprB Superfamily/Carbohydrate-selective porin” (Supplementary Table 4). The gene encoding the Fe-stress marker protein Flavodoxin, *isiB*, was also significantly decreased with Fe-addition (Fig. 3A–C).

#### Estimating *Synechococcus* Fe-status in situ

**Fig. 4:**
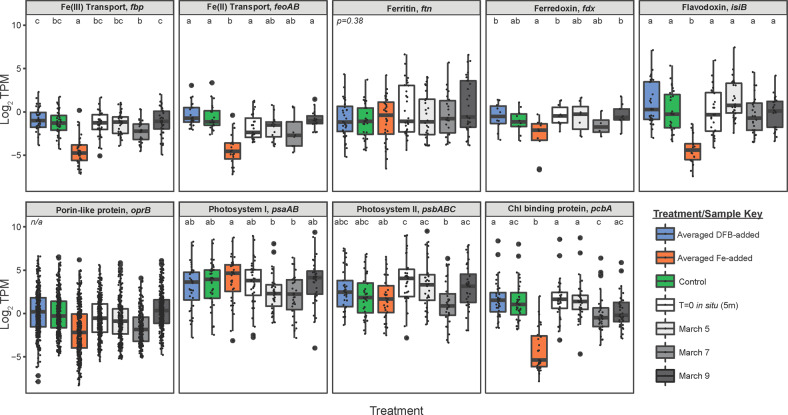
Interpretation of the in situ Fe status of *Synechococcus* communities using the incubations as reference. **A**–**I** Comparison of the transcript abundance of different Fe cycling marker genes across low Fe (averaged DFB-added), high Fe (averaged Fe-added) and in situ surface samples (5–15 m depth). Transcript abundance values are in normalized TPM (log_2_).

To assess the Fe-status of putative *Synechococcus* members in situ, genes indicative of Fe stress were queried and within 4 metatranscriptomes from surface samples (Fig. 4, Supplementary Table 10). Transcript abundances of genes assigned to a ferric binding protein (*fbp*), involved in ABC-transport of Fe(III) species [58], was significantly elevated in the +DFB, control, and *in situ* samples compared to +Fe (Fig. 4, Supplementary Table 11). Likewise, genes assigned an Fe(II) transport system, *feoAB* [58], and flavodoxin (*isiB*), an Fe-stress indicator [10], showed statistically significant similar trends (Fig. 4, Supplementary Table 11). Interestingly, ferredoxin (*fdx*), known to be replaced with flavodoxin under Fe-limitated conditions [10, 59–61], was significantly higher within the +DFB conditions compared to +Fe (p_ad_j = 0.0313) (Fig. 4, Supplementary Table 11). Furthermore, individual *Synecoccocus fdx* harbored on different contigs shows a separation between those elevated under DFB-added conditions (*p* = 0.0003), and those elevated under Fe-added conditions (*p* = 0.0426, Supplementary Fig. 4). Transcripts for genes assigned to the putative Fe-storage protein, ferritin [58, 62], displayed no statistically significant trends across conditions (Fig. 4, Supplementary Table 11). Transcripts for genes involved in electron transport of photosystem I (“PSI”, *psaAB*) were not significantly different between bottle incubations, but were significantly reduced in the March 5th and March 7th in situ samples compared to +Fe conditions (Fig. 4, Supplementary Table 11). Similarly, photosystem II (“PSII”, *psbABC*) genes did not differ significantly between treatments or between the in situ samples compared to the incubations (Fig. 4, Supplementary Table 11). The gene encoding the chlorophyll *a*/*b* binding antennae protein shown to be induced upon Fe-deficiency in cyanobacteria [63], *pcbA*, had significantly elevated transcript abundances under low-Fe conditions and the *in situ* samples compared to the +Fe conditions (Fig. 4, Supplementary Table 11).

### Analysis of other Fe-responsive *Synechococcus* genes

**Fig. 5:**
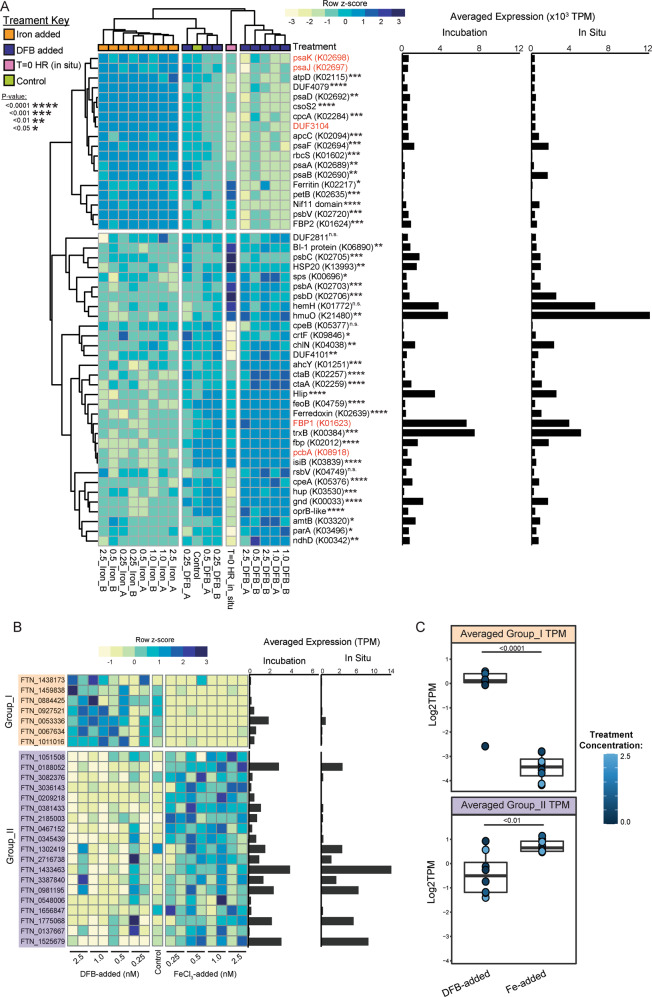
Transcript abundance patterns of genes assigned to *Synechococcus* across Fe availability. **A** The top 50 most abundant genes with the highest variance across Fe-added and DFB-added incubations. Each column is a different bottle from the GRW1 incubation, where “A” and “B” show the result for each biological replicate per treatment. Each value shows the averaged log_2_ normalized transcripts (TPM) across all genes identified as the annotation shown next to each row within the heatmap, scaled by row (z-score, [Observed TPM – mean TPM]/standard deviation) and clustered by sample and gene using a Euclidian distance metric. Full gene names are shown in Supplementary Table 9. *P* values next to each gene were calculated by performing appropriate unpaired *t* tests between log_2_(TPM) values from the +DFB-added incubations versus the +Fe-added incubations. Gene names in red were not statistically analyzed due to having a non-normal distribution and heteroskedasticity. The averaged transcript abundance values for each gene are shown alongside within the Fe incubations (left) and across in situ surface samples (right). **B** Heatmap of individual (un-averaged) *Synechococcus* ferritin separated between low (orange block, “Group I”) and high-Fe (purple block, “Group II”) expressed genes. Averaged TPM values across the incubation experiments and in situ surface samples for each are shown alongside. Phylogenetic placement of each gene is shown in Supplementary Fig. 4. **C** Comparison of the averaged values within each ferritin “Group” heatmap between DFB-added and Fe-added incubations. Each dot represents the averaged value within each incubation bottle, color coded by the level of either DFB or Fe added. P-values were calculated using unpaired t-test with Welch’s correction for Group_I values, and the Mann-Whitney non-parametric test for Group_II values.

Genes with the highest variability in transcript abundances between the +DFB and +Fe incubations, deduced from ranking eigenvalues of each variable across PC2 (Fig. 1B), were queried. Here, the top 50 most abundant *Synechococcus*-like genes ranged in assigned function from Photosynthesis/Energy generation to Fe- and nitrogen-cycling (Supplementary Table 12). Genes encoding PSI components (*psaABDFJK*), F-type H + /Na + -transporting ATPase (*atpD*), carboxysome shell peptide (*csoS2*), phycocyanin alpha chain (*cpcA*), ribulose bisphosphate carboxylase (*rbcS*), fructose-bisphosphate aldolase class-I (FBP2), ferritin, Nif11 domain-like protein, cytochrome *b*_*6*_ (*petB*), and the phycobilisome core linker protein (*apcC*) had significantly elevated transcript abundance across the +Fe incubations compared to the +DFB incubations (Fig. 5A, Supplementary Table 13). Genes involved in PSII function (*psbACD)*, phycoerythrin synthesis (*cpeAB*), light-independent protochlorophyllide reductase (*chlN*), ferredoxin, flavodoxin (*isiB*), chlorophyll *a*/*b* binding antennae protein (*pcbA*), thioredoxin reductase (*trxB*), a high light inducible protein (Hlip), NAD(P)H-quinone oxidoreductase chain 4 (*ndhD*), fructose-bisphosphate aldolase class-I (FBP1), SAM dependent carboxyl methyltransferase (*crtF*), heat shock protein 20 (HSP20), and ammonium transport (*amtB*) were elevated under DFB-added treatments. Fe cycling genes such as the heme utilization gene (*hmuO*), heme a- and o-synthase (*ctaA* and *ctaB*), Fe(II) transport (*feoB*), Fe(III) transport (*fbp*) had significantly higher transcript abundance across the +DFB treatments compared the to +Fe treatments (Fig. 5A, Supplementary Table 13). The heme biosynthesis ferrochelatase gene (*hemH*) trended toward having higher values within the +DFB conditions, but did not have statistically significantly higher transcript abundance in the +DFB relative to the +Fe conditions (Fig. 5A, Supplementary Table 13). Ferritin-like genes in this subset showed on-average higher representation under high-Fe conditions (Fig. 5A, Supplementary Table 13). However, analysis of individual *Synechococcus* ferritin-like transcripts revealed ferritins with higher abundances in the low-Fe treatments and ferritins with higher abundances in the high-Fe treatments (Fig. 5B). These sequences with contrasting patterns are phylogenetically distinct (Supplementary Fig. 5), with the low-Fe ferritin clustering closely (bootstrap > 0.5) with a *Synechococcus* sp. CC9311 reference (“Group_I”) and high-Fe ferritin clustering closely (bootstrap > 0.5) with *Synechococcus* sp. WH8020 and BL107 reference (“Group_II”, Supplementary Fig. 5). Comparison of averaged transcript abundances within each group of ferritin between +DFB and +Fe-added incubations show these patterns are statisticaly significant (Fig. 5C, Supplementary Table 13).

### Read recruitment to genomes of *Synechococcus* isolates

To expand our observations of ecotype-specific *Synechococcus* responses to Fe within the metatranscriptome assembly, and to build linkages to laboratory studies, we competitively mapped to genomes of *Synechococcus* sp. CC9311 (“CC9311”) and *Synechococcus* sp. BL107 (“BL107”). Around 0.40–0.62% of transcripts mapped to CC9311 and 1.13–2.07% of transcripts mapped to BL107 (Supplementary Table 14). Genes of interest from the assembly method were targeted for examination. The analysis revealed 5 ferritins and 3 ferredoxins with differential patterns of representation influenced by Fe availability within the CC9311 genome, whereas BL107 only had 1 ferritin and 1 ferredoxin responsive to Fe (Supplementary Fig. 6 and Supplementary Table 15). Additionaly, *rpoB* transcripts followed similar trends as seen for CC9311-like and BL107-like RpoB (Fig. 1B, Supplementary Fig. 2). In both genomes, there were multiple *oprB*-like porins responsive to Fe. Interestingly, CC9311 had reads mapped to *feoAB* and multiple ABC-transporters, whereas BL107 had few ABC transport components with recruitments and mostly had Fe-uptake porins responsive to Fe availability (Supplementary Fig. 6A). Most genes within the BL107 genome had higher transcript abundances than genes in the CC9311 genome across both the incubations and in situ surface samples (Supplementary Fig. 6B). To confirm the patterns seen here within the GRW1, we also mapped metatranscriptomic reads from GRW2 to these genomes. Read mapping to CC9311 and BL107 genomes from GRW2 metatranscriptomes resulted in 0.48–0.68% and 0.54–0.90% reads mapped, respectively (Supplementary Table 14). Genes of interest (*rpoB*, ferritin, ferredoxins, porins, ABC-type transporters, flavodoxin) within both CC9311 and BL107 genomes showed patterns similar to GRW1 for +Fe *versus* + DFB conditions (Supplementary Fig. 7 and Supplementary Table 15).

## Discussion

Since the seminal work of Martin and colleagues (*e.g.,* [64, 65]), it is clear that Fe constrains marine productivity in many surface oceans [4]. Moreover, subsequent studies have demonstrated the effects of Fe on microbial community diversity—especially for planktonic phototrophs [6, 66, 67]. Using current generation molecular techniques, correlative examinations present evidence that specific populations, with particular genetic capabilities, are distributed across the surface oceans that is in part constrained by Fe-availability [16, 21]. Building on these foundations, the present study provides an experimental approach to diagnose the specific responses of phytoplankton to changes across a gradient of Fe availabilities. Our results demonstrate how the titration of available Fe can be used to both discern community-level Fe-status in the surface-ocean, and to tease apart sub-populations, revealing the persistence and genetic potential of phytoplankton ecotypes. However useful this titration approach was when viewing “bulk” community photosynthetic parameters such as chlorophyll *a* and photosystem II health (Fv/Fm), it did not capture the responses of different microbial community members at high resolution. The employment of metatranscriptomics, however, allowed experimental demonstration of the potential for different *Synechococcus* ecotypes to respond across a gradient of available Fe, illuminating the strategies that different potential ecotypes employ.

We show that bulk photosynthetic biomass and health of the community increased linearly with Fe availability at SOTS, with a pronounced decrease in both photosynthetic biomass and health when DFB was added. This approach has been used previously in field studies to assess the microbial responses to Fe limitation, albeit with coarser (*e.g*., bulk pigment) metrics [68–70]. Analyses of photosynthetic health and flow cytometric cell counts using this approach in the subtropical Pacific Ocean (HNLC region) showed that most cell types (large eukaryotes *versus* cyanobacteria) increased in abundance with increased Fe availability, although cyanobacteria displayed less severe Fe limitation [68]. In the equatorial Pacific Ocean, *Prochlorococcus* exhibited a minimal response to Fe additions whereas large phytoplankton and *Synechococcus* increased in growth [71], leading to the hypothesis of its low-Fe adaptation, which is reflected in its genomic content [16]. Furthermore, culture-based transcriptome responses of *Prochlorococcus* light ecotypes to different Fe availabilities hint towards various mechanisms encoded within the genomes of strains (*e.g.*, Fe transport systems, photosynthetic antennae proteins, Fe-deficiency-induced response genes) that can grow under low Fe concentrations compared to those that cannot [72]. Here, we see similar trends with *Synechococcus*, with an added layer being the presence of putative low- and high-Fe adapted ‘ecotypes’ at SOTS. An expedition in the HNLC, sub-Antarctic Southern Ocean (‘FeCycle’) measured high contributions of cyanobacteria to biotic Fe pools [73], and *Synechococcus* has been shown to be the primary genus (~70%) of cyanobacteria within the SOTS phytoplankton community [74]. It is possible that the seasonal Fe-limitation in the SAZ, combined with periodic inputs of Fe [28] and frequent injections of subtropical water across this region result in a diverse community of *Synechococcus* adapted to high and low Fe conditions. This transcriptomics approach demonstrated the co-occurrence of different Fe-phytoplankton within this region, overlooked by genomic analyses alone, and reflected overlapping niche space derived from episodic exposure, to and/or fluctuations in, Fe availability. That such variability in conditions results in variability in the phytoplankton community has been a central tenet of ocean sciences and microbiology for more than 60 years [19].

Functional analyses further indicated that variable *Synechococcus* types were present and adapted to differing Fe-conditions. Genes involved in Fe-cycling and acquisition were the most responsive to changes in Fe-availability, as many such pathways are controlled by the ferric uptake regulator protein, Fur [75]. Differential transcript abundance analysis of high- and low-Fe treatments highlight a potentially important Fe acquisition mechanism in cyanobacteria that was recently shown to involve a Fe-specific porin [53]. This gene was annotated as an *oprB*-like porin gene with significant sequence similarity to the porin recently identified in Qiu *et al*. 2021. The transcript abundance of this porin-like transporter under both high- and low-Fe conditions, as well as in situ, may provide an advantage to some cyanobacteria, as the passive diffusion through a substrate-specific porin may be an alternative Fe-uptake pathway aside from energy-costly active transport [76, 77]. However, it remains unclear which chemical forms of Fe are transported through the porin, or if the porin may transport other small molecules, as OprB-like proteins mediate sugar uptake in *Pseudomonas aeruginosa* [78] and the cyanobacterium *Nostoc punctiforme* [79]. Moreover, it is thought that a broad spectrum of compounds might make up the organic ligands known to bind Fe in seawater [80] and to be from sources ranging from active production [81] to predation and lysis byproducts [11, 82]. Unsurprisingly, we saw transcripts for genes encoding Fe(III) and Fe(II) active transport systems *fbp* and *feoA* also overrepresented under Fe-limiting conditions. Finally, heme has also been recognized as a potentially significant source of Fe for phytoplankton in the ocean when other forms of Fe are limited [83, 84], and our analysis indicate multiple genes involved in putative heme biosynthesis (*ctaAB, hemH*) and utilization (*hmuO*) processes with transcript abundances overrepresented under Fe-limited incubations and *in situ*. The Heme/hemoglobin/transferrin/lactoferrin TonB-dependent outer membrane receptor, *phuR* [57], had elevated transcript abundances under Fe-limited conditions as well, however it is possible that this gene could be involved in the uptake of other organic Fe sources. Thus, it is possible that these genes may not be directly involved the uptake and utilization of heme as an Fe-source under Fe-limiting conditions, but rather the management of intracellular heme pools and other cellular processes [83], warranting better characterization of the function of heme-cycling genes in cyanobacteria as a response to Fe-limitation. The availability of more resolved Fe-specific pathways from cultured isolate work in the future should allow for a better resolution of the specific Fe-sources these cells are using. While we cannot fully distinguish all forms of Fe preferred by *Synechococcus*, the analyses highlight the diverse mechanisms in which cyanobacteria can acquire Fe at SOTS.

Although we observed more low-Fe adapted *Synechococcus* representation under low-Fe conditions, metatranscriptomes suggested these cells were still exhibiting signatures of Fe-stress. Transcripts for common genetic markers of Fe-stress found to be transcribed under Fe-limiting conditions, namely the chlorophyll binding gene *pcbA* [63], and flavodoxin *isiB* [85–87] were elevated under low-Fe treatments and prevalent *in situ* (Fig. 3 & 4). Paradoxically, ferredoxin, which is known to be replaced by flavodoxin when the cell is Fe-stressed [61, 88, 89], was elevated under low-Fe conditions (Figs. 4F and 5A). A transcriptome study of genome-wide response to Fe-deficiency in *Synechocystis* sp. PCC6803 also found one out of 6 ferredoxin genes 3- to 4-fold up-regulated under Fe-limitation in contrast to the 5 down-regulated ferredoxins [90]. It was found that in *Synechocystis* sp. PCC6803, distinct ferredoxin paralogs played a role in the tolerance to oxidative and metal stress [91], and a unique ferredoxin (Fed2), not involved in photosynthetic electron transport, was shown to be involved in Fe-perception under low-Fe conditions [92]. Further, different ferredoxin paralogs across *Synechococcus* genomes had ecotype-specific patterns across high- and low-Fe oceanic regions [21], suggesting ecotype-specific purposes / repurposing for ferredoxin. Finer scale analysis of each *Synechococcus-*like ferredoxin detected in our analyses showed a similar trend, where a subset of ferredoxin genes had higher representation under high Fe conditions and another subset with higher representation under low Fe conditions, including those most confidently associated with *Synechococcus* CC9311 (Supplementary Fig. 4).

Ferritin is thought to serve as an Fe storage compound in eukaryotes [93] and cyanobacteria [94] when Fe is replete. It has been shown to also be involved in diel regulation of Fe-uptake and recycling and the maintenance of cellular Fe-homeostasis for *Ostreococcus* [95]. In the coastal strain *Synechococcus* sp. CC9311 [96, 97] and *Synechocystis* [98] it is thought to play a role in coping with oxidative stress. Ferritin paralogs also show different abundances across Fe regimes [21], hinting towards ferritins role in multiple cellular processes aside from Fe storage under Fe-replete conditions, as has been shown in diatoms [99]. Furthermore, different *Prochlorococcus* light-ecotypes with varying growth and transcriptome responses under low-Fe concentrations had strain-specific expression of ferritin-like genes, and it was hypothesized that ferritins may serve different functions in different strains depending on their response to Fe-limitation [72]. Here, we see a taxon-specific representation of ferritin-like transcripts, where most are represented under high-Fe conditions but some, clustering phylogenetically with *Synechococcus* sp. CC9311, are more represented under low Fe conditions (Fig. 5B and Supplementary Fig. 5). Indeed, competitive recruitment of the metatranscriptomes to *Synechococcus* sp. CC9311 (a high-nutrient adapted strain) and *Synechococcus* BL107 (an open-ocean/oligotrophic strain) revealed 5 ferritins and 3 ferredoxins with different transcript abundance patterns against Fe-availability from the CC9311 genome whereas BL107 had only 1 ferritin and 1 flavodoxin responsive to Fe (Supplementary Fig. 6). It is unexpected to see a higher representation of ferritin-like genes under low Fe conditions: we posit that CC9311 ferritins may play a role in an overall cellular response to stress for high-Fe adapted strains coping with Fe limitation. These analyses highlight the complex response of high-nutrient adapted CC9311-like strains in comparison to the low-nutrient adapted BL107-like strains with changes in Fe availability: CC9311-like strains use multiple Fe acquisition systems to obtain Fe under both conditions and expresses ferritin even under Fe-limited states to either attempt to store any Fe it takes in or to cope with cellular stress. In contrast, the more streamlined, low-Fe adapted strains, such as BL107 may persist better in low-Fe waters [16]. However, this strategy may place these strains at a disadvantage under short-term Fe increases. Although we cannot fully characterize the *Synechococcus* in our dataset to the strain level, these complex responses to variable Fe conditions point towards different strategies to cope with Fe-limitation, with evidence for both strategies in the surface waters at SOTS.

Overall, by leveraging metatranscriptomes across Fe titrations to interpret *in situ* samples, we captured adaptations to changes in Fe availability of an important group of phytoplankton at SOTS composed of different ecotypes that would have been overlooked in the “bulk” phytoplankton physiological responses. Indeed, altering Fe availability by adding DFB or FeCl_3_ stimulated net chlorophyll *a* and photosystem II health within the bulk community. Since this region is seasonally Fe-limited and encounters episodic inputs of Fe [28], it is possible that high-nutrient adapted “opportunists” respond quickly to these transient pulses, allowing their persistence in this region. Such local adaptation may drive the evolution of clade-specific paralogs of Fe acquisition genes, putative Fe storage genes (ferritins), ferredoxins, and flavodoxins, which have different abundances across varying Fe regimes [21] and could be transcriptionally activated under differing Fe concentrations as suggested by our data. These results warrant better characterization of potential *Synechococcus* Fe-ecotypes and their complex responses to episodic Fe availability in HNLC regions.

## Supplementary information

Supplemental Method

Supplementary Figures

Supplementary Tables

## Data Availability

Raw and processed data used in this study are publicly available through the JGI Data Portal (https://data.jgi.doe.gov) under the Project ID numbers 1260738 and 1260735.
