# Supplementary material for: Bioavailable iron titrations reveal oceanic Synechococcus ecotypes optimized for different iron availabilities
Source: ISME Commun. 2022 Jul 1;2:54. doi: 10.1038/s43705-022-00132-5 (PMC9723758; doi:10.1038/s43705-022-00132-5)
Supplement: Supplementary file 1 — Supplemental Method [file 43705_2022_132_MOESM1_ESM.pdf]

SUPPLEMENTARY METHODS

Bioavailable iron titrations reveal oceanic *Synechococcus* ecotypes optimized for different iron availabilities

Naomi E Gilbert[1], Gary R LeCleir[1], Robert F. Strzepek[2#], Michael J. Ellwood[3], Benjamin S. Twining[4], S. Roux[5], C. Pennacchio[5], Philip W. Boyd[2] and Steven W. Wilhelm[1*]

1. Department of Microbiology, The University of Tennessee, Knoxville, TN 37996 USA
2. Institute for Marine and Antarctic Studies, University of Tasmania, Hobart, Tas. 7004, Australia.
3. Research School of Earth Sciences, Australian National University, Canberra, Australia
4. Bigelow Laboratory for Ocean Sciences, East Boothbay, Maine, USA
5. DOE Joint Genome Institute, Lawrence Berkeley National Laboratory, Berkeley, CA, USA

#. Current Affiliation: Australian Antarctic Program Partnership (AAPP), Institute for Marine and Antarctic Studies, University of Tasmania, Hobart, Tas. 7004, Australia.

Experimental setup and sampling

On-deck iron titration bottle experimental setup

All Fe amendment experiments were done under trace-metal-clean conditions (1). Whole seawater was collected as stated previously using the trace-metal clean pump sampler and dispensed into 2 x 20L Cubitainers® which were acid soaked (reagent-grade hydrochloric, 10%) and rinsed with whole surface seawater. Individual 2L Nalgene™ bottles were cleaned and prepared prior to the incubation by soaking with acid (reagent-grade hydrochloric, 10%) and rinsing three times with seawater. Two Fe incubation experiments were performed on two different dates, March 5th, 2018 (GRW1) and March 14th, 2018 (GRW2). For GRW1, 2L of whole surface water were dispensed into the bottles and amended with 0.25 nM, 0.5 nM, 1.0 nM, or 2.5 nM of either desferrioxamine-B (DFB) or Fe chloride ($FeCl_3$) to chelate available Fe or increase available Fe, respectively. For GRW2, bottles prepared in the same way were amended with either 12.5 nM, 5.0 nM, and 1.25 nM DFB, or 2.5 nM $FeCl_3$. The increase in Fe removal was chosen due to observations made underway. An unamended treatment was included as the control for each experiment. All treatments were performed in duplicate. Bottles were incubated in an on-deck surface seawater flow through incubator at ambient surface temperature (~11° C). To achieve *in situ* light levels (~33% of the incident irradiance) the bottles were covered with neutral density screening.

Chlorophyll a*, photosynthetic efficiency (Fv/Fm) and RNA sample processing*

After 72 h, bottles were destructively sampled for chlorophyll *a*, photosynthetic health (Fv/Fm), and total RNA. For total chlorophyll, 100 mL of water was filtered in duplicate per bottle through a 0.2-µm, 47 mm diameter polycarbonate membrane filter, placed in a clean borosilicate tube with 90% acetone, and stored at 4° C in the dark overnight. The extracted chlorophyll was determined using a Turner designs AU-10 fluorometer (2). Fast Repetition Rate fluorometry was used to determine F_v/F_m and σ_{PSII} using a LIFT-FRR Fluorometer (Soliense, USA). After low light adaptation for ~30 minutes, samples were exposed to 140 flashes of light every 2.5 µsec (saturation sequence) to saturate photosystem II (PSII) and the first stable electron acceptor, QA after which the time interval between flashes was increased exponentially (relaxation sequence) for 90 flashes. Fv/Fm and σPSII were determined from the mean of 200 iterations of the fluorescence induction and relaxation protocol measured at 470 nm. Approximately 1L of water was filtered using a peristaltic pump through 0.2-µm pore-size Sterivex™ filter units and immediately flash frozen and stored in liquid nitrogen in the field, and then at -80° C until further processing.

Bioinformatic and statistical analyses

Raw read processing, assembly, gene calling, and read recruitment

Raw read trimming and filtering to remove adaptor sequences, contaminant sequences, ribosomal RNA, and sequencing spike-ins was done by DOE JGI using BBDuk v38.67 and BBMap v38.84 from the BBtools suite of packages (3, 4). Trimmed reads from all 18 Fe incubation samples of GRW1, including the T=0hr *in situ* sample, were concatenated and assembled ("co-assembled") using MEGAHIT v1.2.9, using the

concatenated reads file as input (5). Open reading frames (ORFs) were called from the combined assembly using the gene finding algorithm MetaGeneMark v3.38 (6, 7). Trimmed reads were mapped to the combined assembly using BBMap v38.84 with default parameters and tabulated using the ORF coordinates with featureCounts v2.0.0 (8). For direct comparison of the *in situ* samples, the trimmed reads from the three libraries were also mapped to the combined assembly from GRW1, and included in tabulation of raw counts. Unless noted otherwise, read mappings across all ORFs detected were normalized using the transcripts-per-million (TPM) approach, as it bypasses inconsistencies of other transcript normalization approaches (9).

Targeted analyses of the DNA-directed RNA polymerase gene and phylogeny

To identify and compare the relative activity of prokaryotic and eukaryotic members within the community to Fe availability, we screened for the DNA-directed RNA polymerase beta subunit (*RPB1,rpoB*) sequences were screened for and their transcript abundances queried as a constitutively expressed housekeeping marker (10-14). Proteins from the GRW1 assembly were queried against a reference protein database (KEGG Orthology, k04043 and k03006) using DIAMOND BLASTp with the --sensitive option and an e-value cutoff of e^{-10} (15). The resulting protein hits were clustered at 95% amino acid similarity using CD-HIT (16) to reduce the dataset from 14139 to 8757 candidates. Clustered sequences were then queried against the NCBI non-redundant database using DIAMOND BLASTp as previously stated, and the top hits for each sequence were manually inspected to include only RpoB or RPB1 proteins. Phylogenetic analysis of the final RpoB/RPB1 hits was done by aligning reference tree sequences with

RpoB/RPB1 hits of ≥1200 aa length in MEGA7 (17) using ClustalW (18). For further exploration of individual cyanobacterial-like RpoB sequences, un-clustered RpoB hits were used to construct a separate tree containing all eukaryotic and prokaryotic references, expect more *Synechococcus* RpoB reference sequences were added for better resolution of this group (Supplementary Table 2). The separate maximum-likelihood trees were constructed in PhyML (19) with the LG model, and the Shimodaira-Hasegawa (SH)-like approximate likelihood ratio test (aLRT-SH-like) to calculate likelihood ratios and branch support. Hits >1200 aa were used within the base PhyML tree. Then, hits <1200 aa were placed on the base tree using pplacer with the empirical frequency type parameter (20) and the resulting tree was visualized and annotated using iTOL v.4 (21).

Functional and taxonomic annotation of Synechococcus-*like sequences*

Further functional annotation of the ORFs was done using eggNOG-mapper v2.0.1 with default parameters (22). To better characterize bacterial Fe-responsive elements like Fe transporters, regulators, and siderophore acquisition genes, the HMM-based program FeGenie was implemented using the assembled contigs as input with default parameters (23). Genes were taxonomically assigned using the “best LAST hit” procedure by the IMG pipeline (4, 24). Because of discrepancies across taxonomic annotators of functional genes in metagenomes, only ORFs annotated as *Synechococcus* using this tool and *via* eggNOG-mapper*’s Best Tax Level*, were retained. A phylogenetic tree was constructed for representative ferritin-like sequences detected to have disparate patterns of transcript abundance with Fe availability using

the same approach outlined above for RpoB/RPB1, except ferritin-like sequences from *Synechococcus* isolate genome sequences were downloaded from NCBI (June 2021) for reference tree building, and putative *Synechococcus* ferritin sequences >190 aa were used in the base tree (available in Supplementary Table 3).

Whole genome analysis of representative Synechococcus isolates

Annotated coding sequences from whole sequenced genomes of *Synechococcus* sp. CC9311 (NCBI accession: CP000435.1) and *Synechococcus* BL107 (NCBI accession: NZ_DS022298.1) were downloaded from NCBI on June 12th, 2021. These two isolates were chosen based on high sequence similarity of *rpoB* and ferritin-like sequences within the assembly, deduced from the taxonomic annotation and phylogenies (Supplementary Figures 1 and 4). They also represent *Synechococcus* species isolated from divergent trophic statuses: *Synechococcus* sp. CC9311 was isolated from the coastal California current (Pacific Ocean) and is known as a coastal, opportunist strain whereas *Synechococcus* BL107 was isolated from Blanes Bay in the Mediterranean Sea and is known as an open-ocean, specialist strain (25). The coding sequences of both genomes were concatenated and using as a reference for read recruitment. Trimmed reads from the GRW1 experiment, GRW2 experiment and *in situ* metatranscriptome libraries were mapped to the concatenated reference using default parameters in CLC Genomics Workbench version 21.0.4 (Qiagen), expect a 85% similarity fraction over a 90% read length fraction threshold was set to stringently assign reads. Reads mapping redundantly to both genomes were not considered. Resulting reads were normalized using the TPM method.

Statistical Analyses

To compare the transcript abundances across genes of interest, data within the DFB-added (0.25 nM – 2.5 nM DFB) and Fe-added (0.25 nM – 2.5 nM $FeCl_3$) incubations were grouped to test the transcriptomic effect of stressing the community for iron (+DFB) or replenishing the community with iron (+Fe) regardless of concentration. Here, log2-transformed TPMs for each gene were tested for normal distribution using the D'Agostino-Pearson omnibus test and the Kolmogorov-Smirnov test. The F-test was used to test for the equality of two variances. If the data were normally distributed and had equal variance, an unpaired two-tailed t-test was used to determine significance. If the data were not normally distributed, an unpaired t-test with Welch's correction was used to determine significance. If the data had non-normal distribution, the Mann-Whitney non-parametric test was used to determine significance. To compare normalized transcript abundances for genes of interest between DFB-added incubations, the D'Agostino-Pearson omnibus test and the Kolmogorov-Smirnov tests were used to test for normality, and the Brown-Forsythe test and Bartlett's test were used to test for the equality of two variances. If the data for each gene had a normal distribution, the ordinary one-way ANOVA was used, with Tukey's HSD correction for post-hoc multiple comparisons. If the data had non-normal distribution, the non-parametric Kruskal Wallis test was used, with Dunn's correction for post-hoc multiple comparisons. The data for the *oprB*-like gene was not statistically analyzed due to both non-normal distribution and heteroskedasticity.

References

1. Bruland KW, Franks RP, Knauer GA, Martin JH. Sampling and analytical methods for the determination of copper, cadmium, zinc, and nickel at the nanogram per liter level in sea water. Anal Chim Acta. 1979;105:233-45.
2. Welschmeyer NA. Fluorometric analysis of chlorophyll a in the presence of chlorophyll b and pheopigments. Limnol Oceanogr. 1994;39(8):1985-92.
3. Bushnell B. BBMap: a fast, accurate, splice-aware aligner. Lawrence Berkeley National Lab.(LBNL), Berkeley, CA (United States); 2014.
4. Clum A, Huntemann M, Bushnell B, Foster B, Foster B, Roux S, et al. DOE JGI Metagenome Workflow. Msystems. 2021;6(3):e00804-20.
5. Li D, Liu C-M, Luo R, Sadakane K, Lam T-W. MEGAHIT: an ultra-fast single-node solution for large and complex metagenomics assembly via succinct de Bruijn graph. Bioinformatics. 2015;31(10):1674-6.
6. Zhu W, Lomsadze A, Borodovsky M. Ab initio gene identification in metagenomic sequences. Nucleic Acids Res. 2010;38(12):e132-e.
7. Besemer J, Borodovsky M. Heuristic approach to deriving models for gene finding. Nucleic Acids Res. 1999;27(19):3911-20.
8. Liao Y, Smyth GK, Shi W. featureCounts: an efficient general purpose program for assigning sequence reads to genomic features. Bioinformatics. 2014;30(7):923-30.
9. Wagner GP, Kin K, Lynch VJ. Measurement of mRNA abundance using RNA-seq data: RPKM measure is inconsistent among samples. Theory Biosci. 2012;131(4):281-5.
10. Ng YK, Hewavitharana AK, Webb R, Shaw PN, Fuerst JA. Developmental cycle and pharmaceutically relevant compounds of Salinispora actinobacteria isolated from Great Barrier Reef marine sponges. Applied Microbiology and Biotechnology. 2013;97(7):3097-108.
11. Fortunato CS, Crump BC. Microbial gene abundance and expression patterns across a river to ocean salinity gradient. PLoS One. 2015;10(11):e0140578.
12. Bagchi S, Lamendella R, Strutt S, Van Loosdrecht MCM, Saikaly PE. Metatranscriptomics reveals the molecular mechanism of large granule formation in granular anammox reactor. Scientific Reports. 2016;6(1):28327.
13. Jones DS, Flood BE, Bailey JV. Metatranscriptomic insights into polyphosphate metabolism in marine sediments. ISME J. 2016;10(4):1015-9.
14. Satinsky BM, Crump BC, Smith CB, Sharma S, Zielinski BL, Doherty M, et al. Microspatial gene expression patterns in the Amazon River Plume. PNAS. 2014;111(30):11085-90.
15. Buchfink B, Xie C, Huson DH. Fast and sensitive protein alignment using DIAMOND. Nat Methods. 2015;12(1):59-60.
16. Fu L, Niu B, Zhu Z, Wu S, Li W. CD-HIT: accelerated for clustering the next-generation sequencing data. Bioinformatics. 2012;28(23):3150-2.
17. Kumar S, Stecher G, Tamura K. MEGA7: molecular evolutionary genetics analysis version 7.0 for bigger datasets. Mol Biol Evol. 2016;33(7):1870-4.
18. Thompson JD, Gibson TJ, Higgins DG. Multiple sequence alignment using ClustalW and ClustalX. Curr Protoc Bioinform. 2003(1):2.3. 1-2.3. 22.

19. Guindon S, Dufayard J-F, Lefort V, Anisimova M, Hordijk W, Gascuel O. New algorithms and methods to estimate maximum-likelihood phylogenies: assessing the performance of PhyML 3.0. Syst Biol. 2010;59(3):307-21.
20. Matsen FA, Kodner RB, Armbrust EV. pplacer: linear time maximum-likelihood and Bayesian phylogenetic placement of sequences onto a fixed reference tree. BMC Bioinf. 2010;11(1):1-16.
21. Letunic I, Bork P. Interactive Tree Of Life (iTOL) v4: recent updates and new developments. Nucleic Acids Res. 2019;47(W1):W256-W9.
22. Huerta-Cepas J, Forslund K, Coelho LP, Szklarczyk D, Jensen LJ, Von Mering C, et al. Fast genome-wide functional annotation through orthology assignment by eggNOG-mapper. Mol Biol Evol. 2017;34(8):2115-22.
23. Garber AI, Nealson KH, Okamoto A, McAllister SM, Chan CS, Barco RA, et al. FeGenie: a comprehensive tool for the identification of iron genes and iron gene neighborhoods in genome and metagenome assemblies. Front Microbiol. 2020;11:37.
24. Kiełbasa SM, Wan R, Sato K, Horton P, Frith MC. Adaptive seeds tame genomic sequence comparison. Genome Res. 2011;21(3):487-93.
25. Dufresne A, Ostrowski M, Scanlan DJ, Garczarek L, Mazard S, Palenik BP, et al. Unraveling the genomic mosaic of a ubiquitous genus of marine cyanobacteria. Genome Biol. 2008;9(5):R90.
