# Supplementary material for: Bioavailable iron titrations reveal oceanic Synechococcus ecotypes optimized for different iron availabilities
Source: ISME Commun. 2022 Jul 1;2:54. doi: 10.1038/s43705-022-00132-5 (PMC9723758; doi:10.1038/s43705-022-00132-5)
Supplement: Supplementary file 2 — Supplementary Figures [file 43705_2022_132_MOESM2_ESM.pdf]

SUPPLEMENTARY FIGURES

Bioavailable iron titrations reveal oceanic *Synechococcus* ecotypes optimized for different iron availabilities

Naomi E Gilbert[1], Gary R LeCleir[1], Robert F. Strzepek[2#], Michael J. Ellwood[3], Benjamin S. Twining[4], S. Roux[5], C. Pennacchio[5], Philip W. Boyd[2] and Steven W. Wilhelm[1*]

1. Department of Microbiology, The University of Tennessee, Knoxville, TN 37996 USA
2. Institute for Marine and Antarctic Studies, University of Tasmania, Hobart, Tas. 7004, Australia.
3. Research School of Earth Sciences, Australian National University, Canberra, Australia
4. Bigelow Laboratory for Ocean Sciences, East Boothbay, Maine, USA
5. DOE Joint Genome Institute, Lawrence Berkeley National Laboratory, Berkeley, CA, USA

#. Current Affiliation: Australian Antarctic Program Partnership (AAPP), Institute for Marine and Antarctic Studies, University of Tasmania, Hobart, Tas. 7004, Australia.

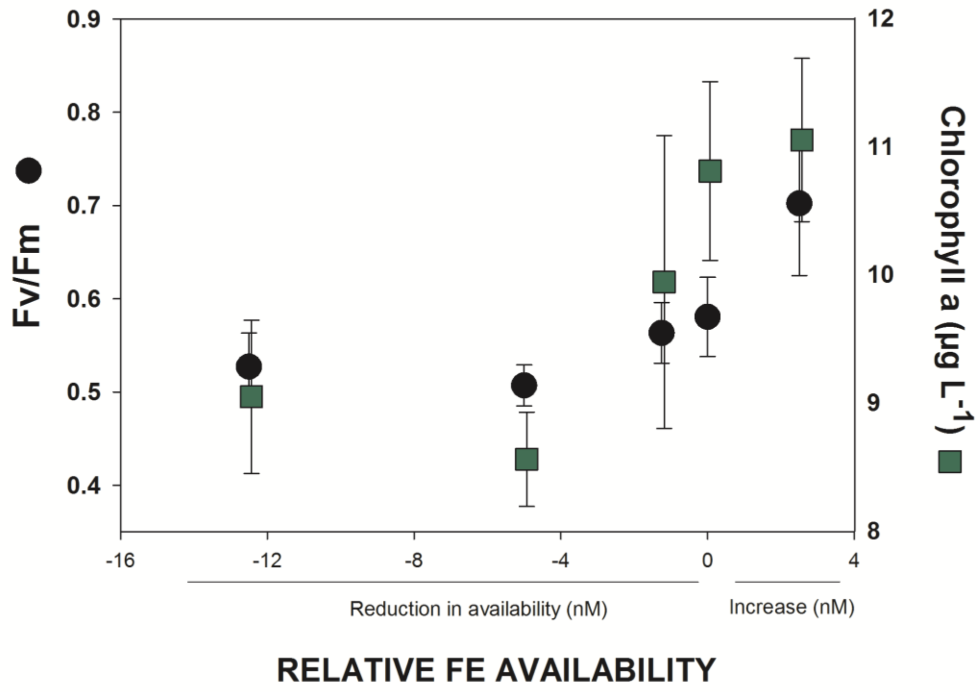

Supplementary Figure 1. Physiological results of the bulk photosynthetic community within the second growout experiment (GRW2). Total phytoplankton biomass estimates (chlorophyll *a* concentrations, green boxes) and photosynthetic efficiency (F_v/F_m, ratio of variable fluorescence to maximal fluorescence, black circles) as a function of relative Fe availability. Fe was reduced by the addition of 12.5 nM, 5.0 nM, or 1.25 nM DFB and Fe was increased by the addition of 2.5 nM $FeCl_3$. Results are shown as the means ± error of technical duplicates, averaged between duplicate bottle.

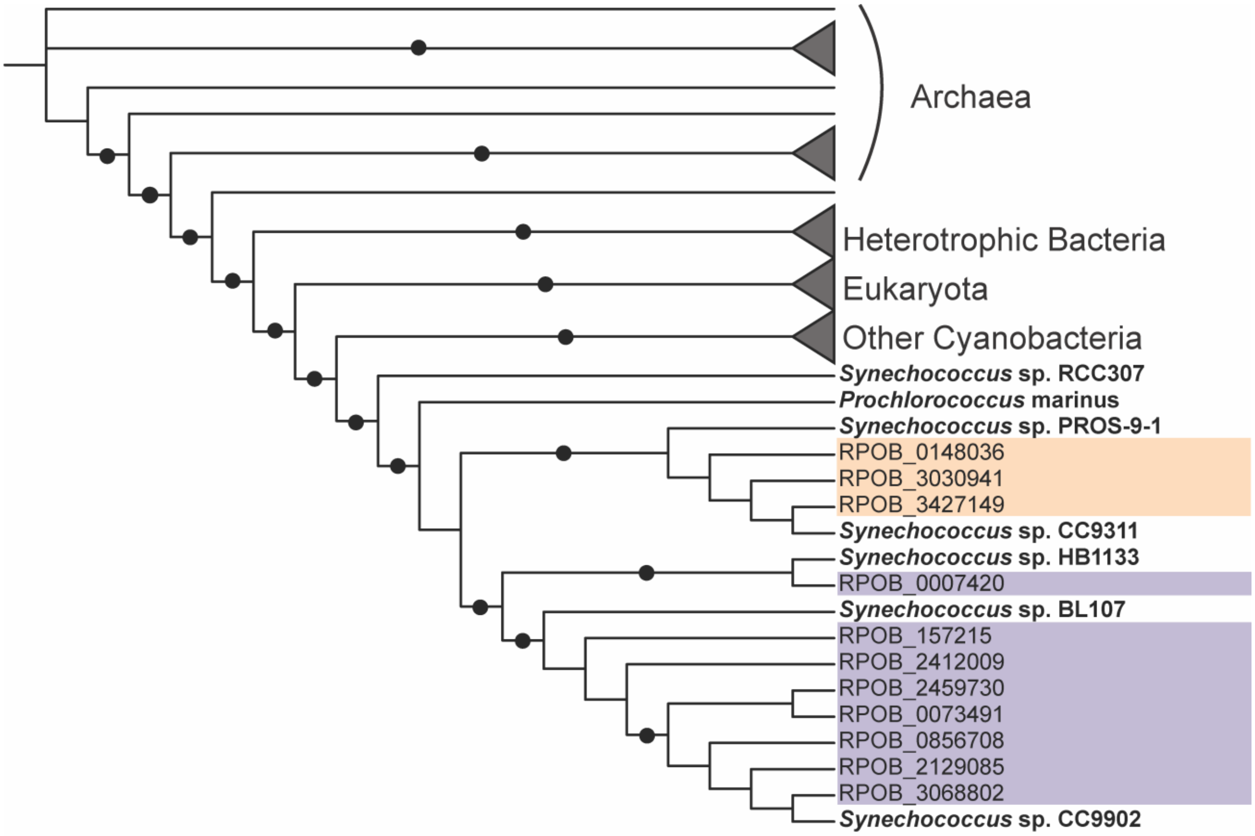

Supplementary Figure 2. Maximum likelihood placement of individual *Synechococcus* RpoB protein sequences, including all eukaryotic and prokaryotic reference RpoB/RPB1 proteins (collapsed triangles). Reference proteins are shown in bold font. Black dots represent bootstrap values > 0.5. Orange and purple blocks correspond to the heatmap shown in Figure 2B.

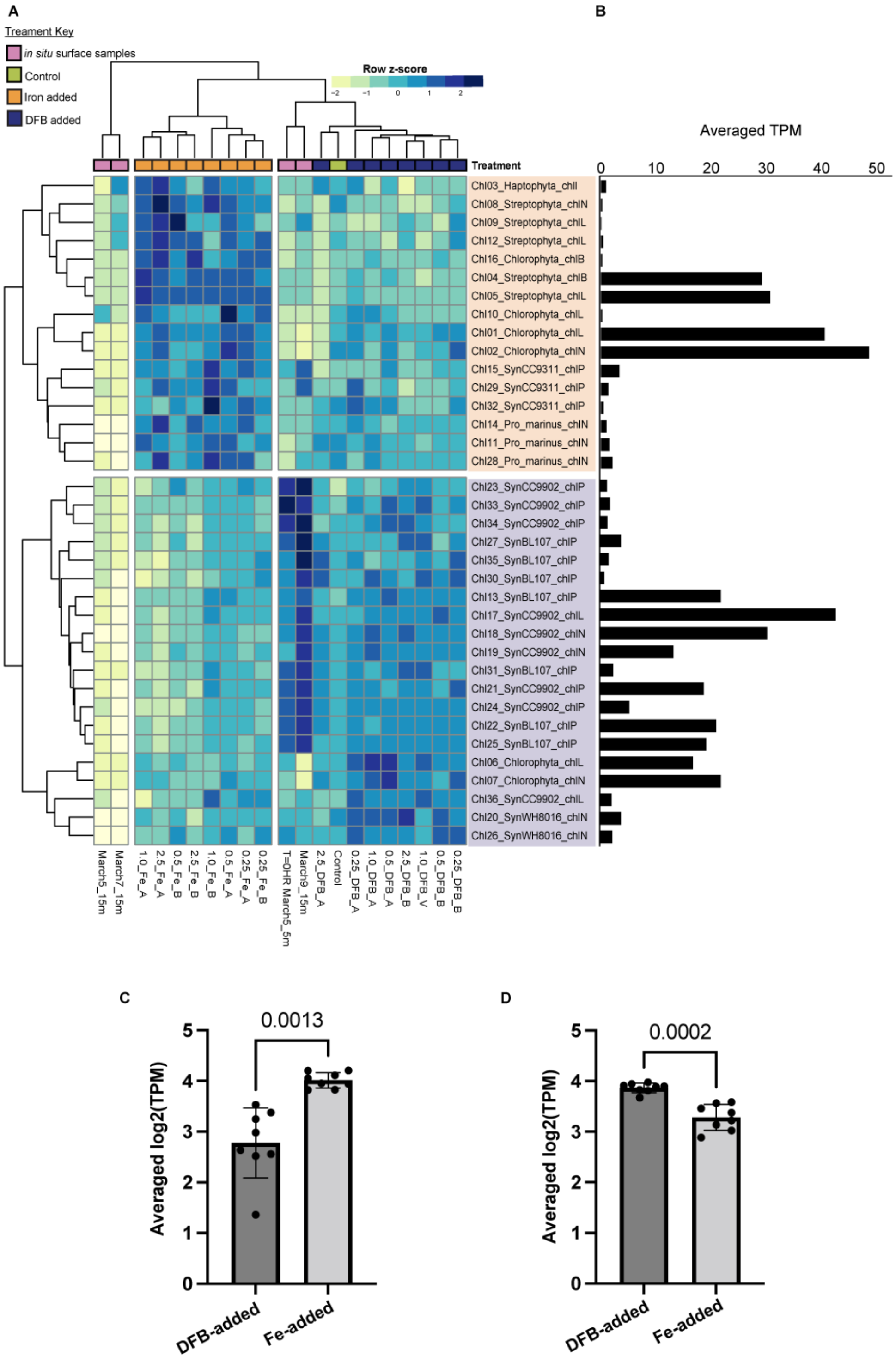

Supplementary Figure 3. Transcript abundance patterns of eukaryotic and cyanobacteria chlorophyll biosynthesis genes (*chlB, chlI, chlL, chlN, chlP*) across Fe and *in situ* surface samples. A) Row-scaled (z-score, [Observed TPM – mean TPM]/standard deviation) normalized transcript abundance values, clustered by gene and sample using the Euclidean distance metric. Each column is a different bottle from

the GRW1 incubation, where “A” and “B” show the result for each biological replicate per treatment. B) Average transcript abundance values (TPM) of each gene across both the incubations and *in situ* samples shown within the heatmap. Row names correspond to gene IDs in Supplementary Table 4). C) Chlorophyll biosynthesis genes with transcripts overrepresented in the high-Fe/Fe-added conditions (orange shaded genes in A). Each bar represents the averaged log2(TPM) of genes within the orange-shaded region in A, with each treatment bottle shown as individual points within each bar. The p-value was calculated using the unpaired t-test with Welch’s correction. D) Chlorophyll biosynthesis genes with transcripts overrepresented in the low-Fe/DFB-added conditions (purple shaded genes in A). Each bar represents the averaged log2(TPM) of genes within the purple-shaded region in A, with each treatment bottle shown as individual points within each bar. The p-value was calculated using the unpaired t-test with Welch’s correction.

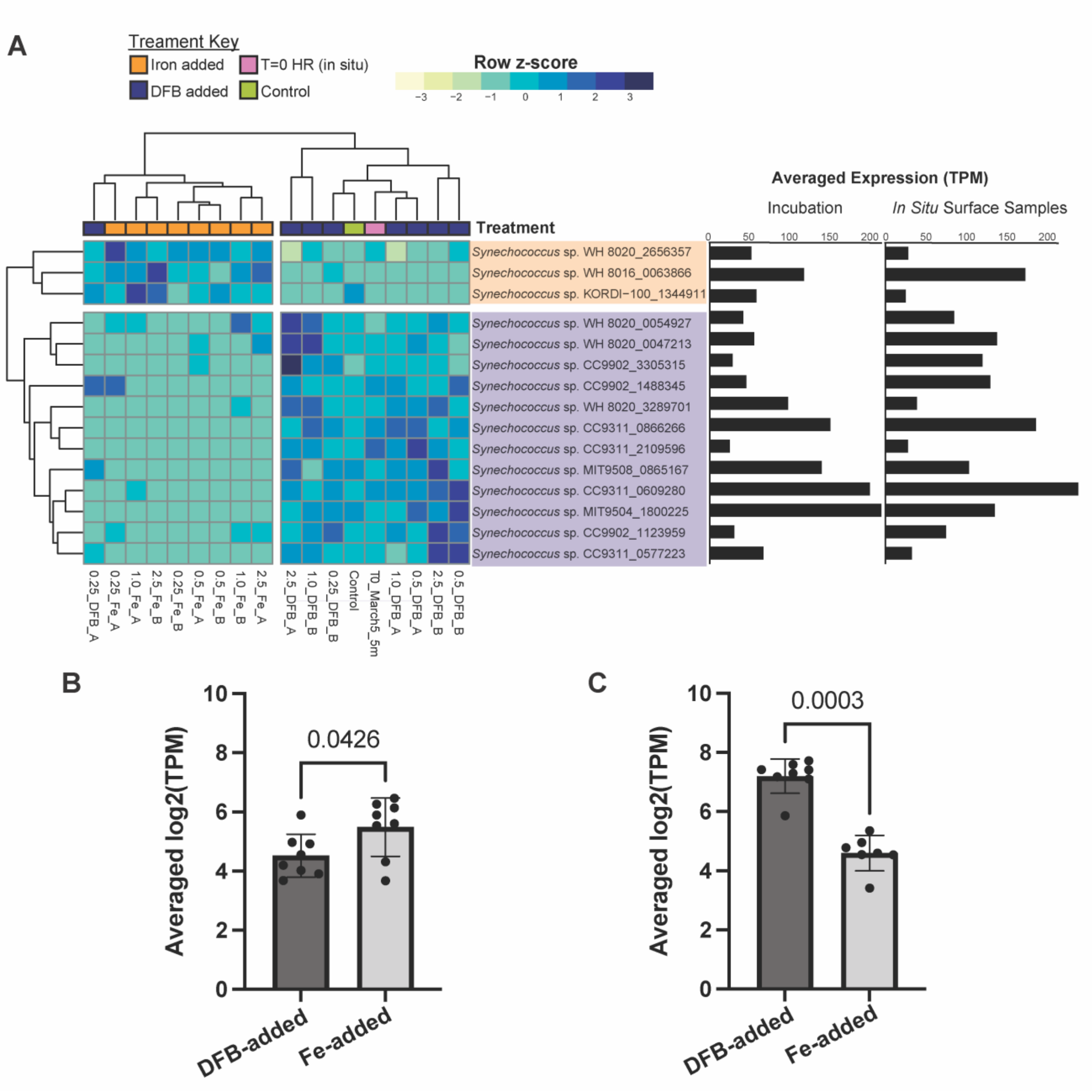

Supplementary Figure 4. Differentiation of high-Fe and low-Fe ferredoxin gene transcripts within the GRW1 assembly. A) Heatmap of individual ferredoxin genes assigned to *Synechococcus*. The row names show the best LAST taxonomic assignment of each gene, followed by the gene ID. Row-scaled TPM (z-score, [Observed TPM – mean TPM]/standard deviation) values are shown in the heatmap and clustered using a Euclidean distance metric. Each column is a different bottle from the GRW1 incubation, where “A” and “B” show the result for each biological replicate per treatment. Averaged transcript abundance (TPM) of each gene across the incubations and *in situ* are shown alongside. B) Ferredoxin genes with transcripts overrepresented in the high-Fe/Fe-added conditions (orange shaded genes in A). Each bar represents the averaged log2(TPM) of

genes within the orange-shaded region in A, with each treatment bottle shown as individual points within each bar. The p-value was calculated using an unpaired t-test. C) Ferredoxin genes with transcripts overrepresented in the low-Fe/DFB-added conditions (purple shaded genes in A). Each bar represents the averaged log2(TPM) of genes within the purple-shaded region in A, with each treatment bottle shown as individual points within each bar. An exact p-value was calculated using the Mann-Whitney test due to non-normal distribution of this data comparison.

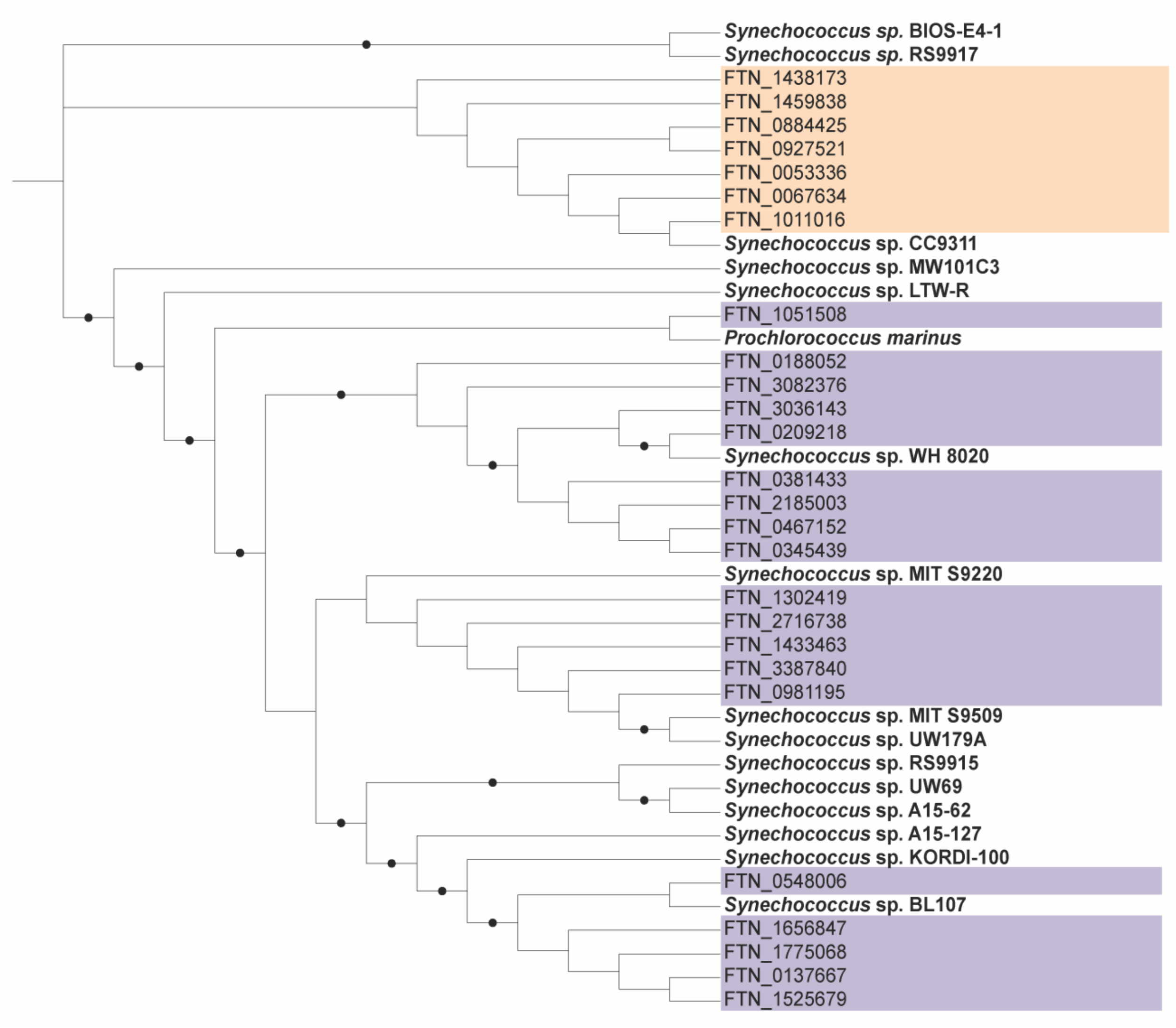

Supplementary Figure 5. Maximum likelihood placement of individual *Synechococcus* (and one *Prochlorococcus,* FTN_1051508) ferritin protein sequences. Reference proteins are shown in bold font. Black dots represent bootstrap values > 0.5. Orange and purple blocks correspond to the heatmap shown in Figure 5B.

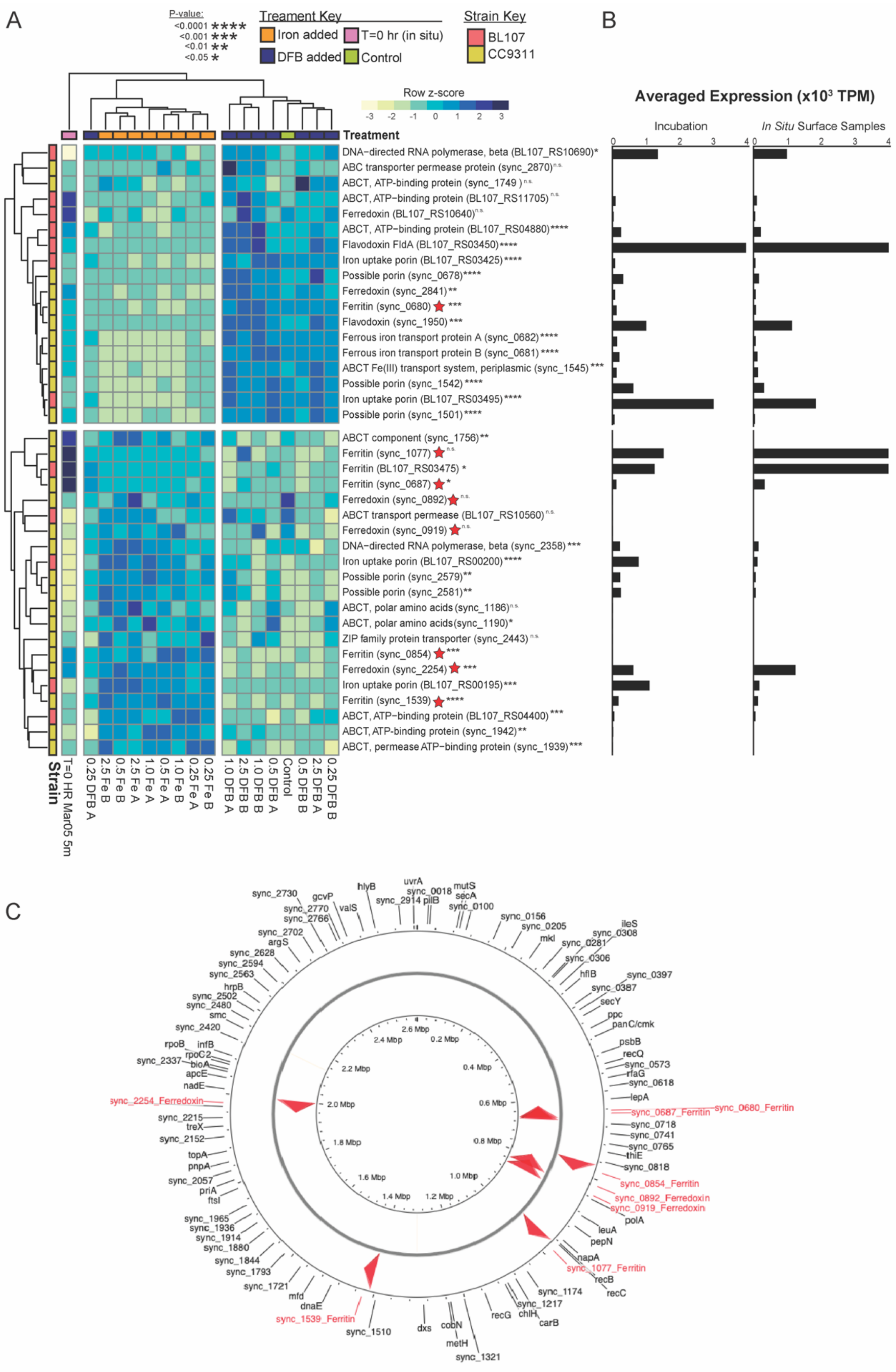

A
P-value:
<0.0001 ****
<0.001 ***
<0.01 **
<0.05 *
Treament Key
Iron added
T=0 hr (in situ)
DFB added
Control
Strain Key
BL107
CC9311
B
Row z-score
-3 -2 -1 0 1 2 3
Treatment
DNA-directed RNA polymerase, beta (BL107_RS10690)*
ABC transporter permease protein (sync_2870) n.s.
ABCT, ATP-binding protein (sync_1749) n.s.
ABCT, ATP-binding protein (BL107_RS11705) n.s.
Ferredoxin (BL107_RS10640) n.s.
ABCT, ATP-binding protein (BL107_RS04880)****
Flavodoxin FldA (BL107_RS03450)****
Iron uptake porin (BL107_RS03425)****
Possible porin (sync_0678)****
Ferredoxin (sync_2841)**
Ferritin (sync_0680) ***
Flavodoxin (sync_1950)***
Ferrous iron transport protein A (sync_0682)****
Ferrous iron transport protein B (sync_0681)****
ABCT Fe(III) transport system, periplasmic (sync_1545)***
Possible porin (sync_1542)****
Iron uptake porin (BL107_RS03495)****
Possible porin (sync_1501)****
ABCT component (sync_1756)**
Ferritin (sync_1077) n.s.
Ferritin (BL107_RS03475) *
Ferritin (sync_0687) *
Ferredoxin (sync_0892) n.s.
ABCT transport permease (BL107_RS10560) n.s.
Ferredoxin (sync_0919) n.s.
DNA-directed RNA polymerase, beta (sync_2358)***
Iron uptake porin (BL107_RS00200)****
Possible porin (sync_2579)**
Possible porin (sync_2581)**
ABCT, polar amino acids (sync_1186) n.s.
ABCT, polar amino acids(sync_1190)*
ZIP family protein transporter (sync_2443) n.s.
Ferritin (sync_0854) ***
Ferredoxin (sync_2254) ***
Iron uptake porin (BL107_RS00195)***
Ferritin (sync_1539) ****
ABCT, ATP-binding protein (BL107_RS04400)***
ABCT, ATP-binding protein (sync_1942)**
ABCT, permease ATP-binding protein (sync_1939)***
Strain
T=0 HR Mar05 5m
0.25 DFB A
2.5 Fe B
0.5 Fe B
2.5 Fe A
1.0 Fe A
0.5 Fe A
1.0 Fe B
0.25 Fe A
0.25 Fe B
1.0 DFB A
2.5 DFB B
1.0 DFB B
0.5 DFB A
Control
0.5 DFB B
2.5 DFB A
0.25 DFB B
Averaged Expression (x10³ TPM)
Incubation
In Situ Surface Samples
0 1 2 3 4
C
2.6 Mbp
0.2 Mbp
0.4 Mbp
0.6 Mbp
0.8 Mbp
1.0 Mbp
1.2 Mbp
1.4 Mbp
1.6 Mbp
1.8 Mbp
2.0 Mbp
2.2 Mbp
2.4 Mbp
sync_2254_Ferredoxin
sync_0687_Ferritin
sync_0680_Ferritin
sync_0854_Ferritin
sync_0892_Ferredoxin
sync_0919_Ferredoxin
sync_1077_Ferritin
sync_1539_Ferritin
uvrA
hlyB
mutS
secA
pilB
gcvP
valS
argS
hrpB
smc
rpoB
infB
rpoC2
bioA
apcE
nadE
treX
topA
pnpA
priA
ftsI
mfd
dnaE
dxs
cobN
metH
recG
chlH
carB
recB
recC
napA
pepN
leuA
polA
thiE
lepA
rfaG
recQ
psbB
panC/cmk
ppc
secY
hflB
mkl
ileS

Supplementary Figure 6. Targeted genome analysis and read recruitment to *Synechococcus* sp. CC9311 (Genbank accession: CP000435.1) and *Synechococcus* sp. BL107 (Genbank accession: NZ_DS022298.1). A) Heatmap displaying genes of interest involved in Fe metabolism and Fe status marker genes comparing competitive read recruitment to each strain (row annotation). Normalized transcript abundance values are scaled by row (z-score, [Observed TPM – mean TPM]/standard deviation). Each column is a different bottle from the GRW1 incubation, where “A” and “B” show the result for each biological replicate per treatment. Red stars represent genes highlighted in red within the CC9311 genome. P-values indicated by asterisk show whether or not the mean transcript values across bottles between +DFB-added and +Fe-added incubations are significantly different using either unpaired t-tests, unpaired t-test with Welch’s correction, or Mann-Whitney tests (see Supplementary Methods for details). B) Averaged transcript (TPM) abundances of each gene across the incubations and *in situ* surface samples (March05_5m, March05_15, March07_15m, March18_15m). C) Annotated genome of *Synechococcus* sp. CC9311. Genes highlighted in red correspond to genes with red stars in Supplementary Figure 5A. The genome map was constructed in http://cgview.ca/ .

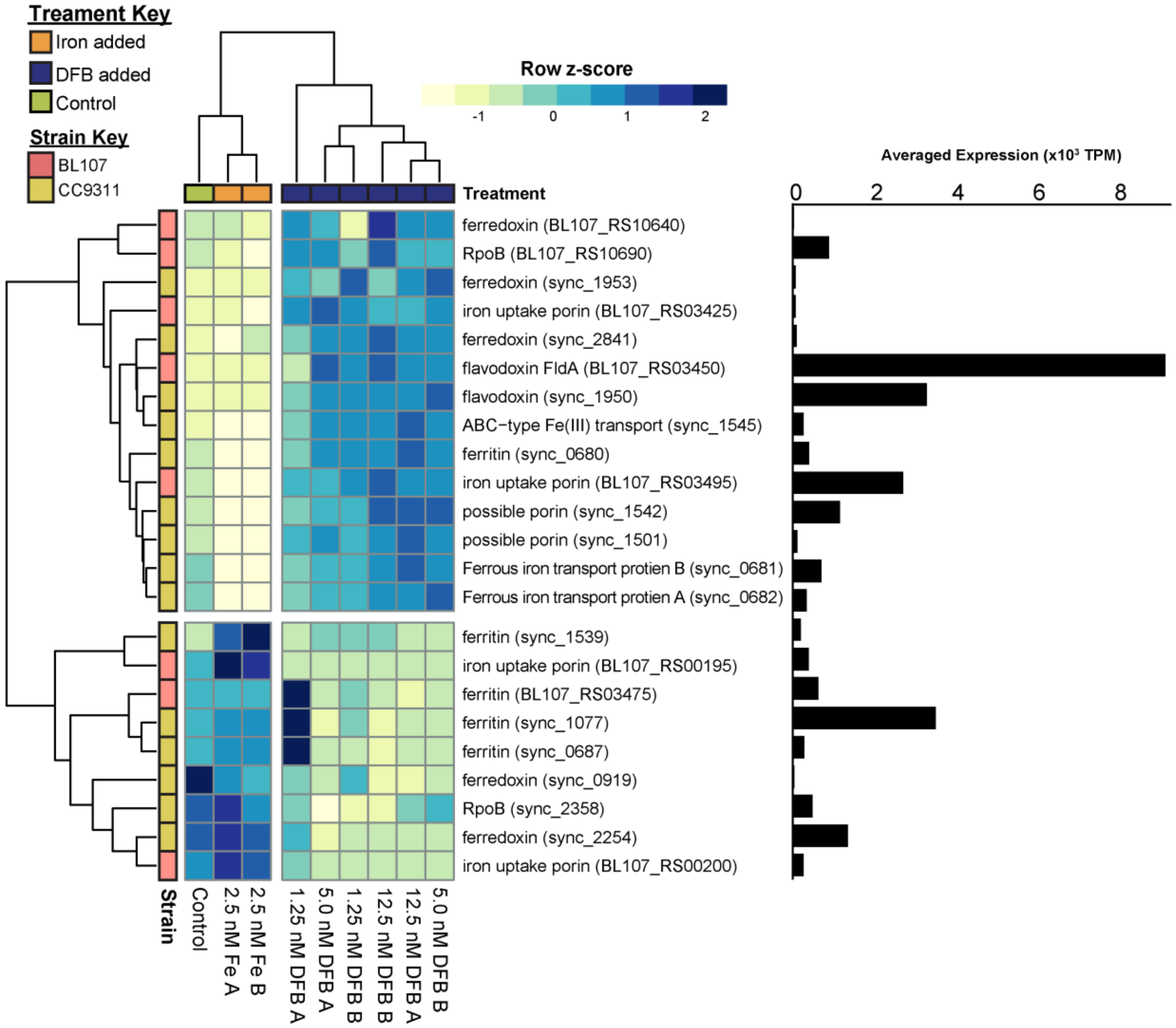

Supplementary Figure 7. Read recruitment of a second DFB and Fe amendment incubation ("GRW2") to *Synechococcus* sp. CC9311 and *Synechococcus* sp. BL107 reference genomes. Each column is a different bottle from the GRW2 incubation, where "A" and "B" show the result for each biological replicate per treatment. Normalized transcript abundance values (TPM) are scaled by row (z-score, [Observed TPM – mean TPM]/standard deviation). Averaged transcript abundances (TPM) for each gene are shown on the left hand bar plot.
